# Comprehensive study of α-terpineol-loaded oil-in-water (O/W) nanoemulsion: interfacial property, formulation, physical and chemical stability

**DOI:** 10.1038/s41538-021-00113-3

**Published:** 2021-11-15

**Authors:** Lorena de Oliveira Felipe, Juliano Lemos Bicas, Meryem Bouhoute, Mitsutoshi Nakajima, Marcos A. Neves

**Affiliations:** 1 Graduate School of Life and Environmental Sciences, University of Tsukuba, 1-1-1 Tennodai, Tsukuba, Ibaraki 305-0006 Japan; 2 School of Food Engineering, Department of Food Science, University of Campinas, Rua Monteiro Lobato, 80. CEP: 13083-862. Campinas–São Paulo, São Paulo, Brazil; 3 Alliance for Research on the Mediterranean and North Africa (ARENA), University of Tsukuba, 1-1-1 Tennodai, Tsukuba, Ibaraki 305-0006 Japan; 4 Faculty of Life and Environmental Sciences, University of Tsukuba, 1-1-1 Tennodai, Tsukuba, Ibaraki 305-0006 Japan

**Keywords:** Nanoscale materials, Engineering

## Abstract

In this study, the interfacial ability of α-terpineol (α-TOH) was reported, followed by its trapping into oil-in-water (O/W) nanoemulsion as active-ingredient and the long-term observation of this nanosystem influenced by the storage-time (410-days) and temperature (5, 25, 50 °C). The results indicated that the α-TOH can reduce the interfacial tension on the liquid-liquid interface (Δ*G*°_m_ = −1.81 KJ mol^−1^; surface density = 8.19 × 10^−6 ^mol m^−2^; polar head group area = 20.29 Å^2^), in the absence or presence of surfactant. The O/W nanoemulsion loaded with a high amount of α-TOH (90 mg mL^−1^; 9α-TOH-NE) into the oil phase was successfully formulated. Among the physical parameters, the mean droplet diameter (MDD) showed a great thermal dependence influenced by the storage-temperature, where the Ostwald ripening (OR) was identified as the main destabilizing phenomena that was taking place on 9α-TOH-NE at 5 and 25 °C along with time. Despite of the physical instability, the integrity of both nanoemulsion at 5 °C and 25 °C was fully preserved up to 410th day, displaying a homogeneous and comparable appearance by visual observation. On contrary, a non-thermal dependence was found for chemical stability, where over 88% of the initial amount of the α-TOH nanoemulsified remained in both 9α-TOH-NE at 5 and 25 °C, up to 410th day. Beyond the key data reported for α-TOH, the importance of this research relies on the long-term tracking of a nanostructured system which can be useful for scientific community as a model for a robust evaluation of nanoemulsion loaded with flavor oils.

## Introduction

Functional ingredients play a crucial role in the food industry. While vitamins and nutraceuticals boost the nutritional value; flavors and colors improve the sensorial quality and food-perception, impacting the food-acceptance by consumers. However, the incorporation of these ingredients into food-products, most of times, implies many challenges due to some features among them: (i) sensitivity to the surrounding environment, and (ii) hydrophobicity of their molecules^1^. Therefore, the properties of these compounds can be easily lost. Frequently, this degradation has moisture-, light- and thermal-dependency, leading to oxidation. Consequently, off-flavors may appear, compromising the food-acceptance. Volatilization also occurs due to temperature degradation of thermolabile molecules. In addition, food products are mostly water-based. Thus, the incorporation of lipophilic compounds such as flavor oils into the aqueous matrix is technically challenging. To overcome these difficulties, trapping sensitive and hydrophobic nutrients through edible emulsion delivery systems is frequently considered as a feasible option^2^.

Emulsions comprise a mixture of two immiscible liquids. One is the water phase and another one is the lipid fraction. Emulsions are classified as macro-, micro- and nanoemulsions based on the droplet size, surface area, and polydispersity index. Amid them, water-in-oil (W/O) or oil-in-water (O/W) nanoemulsion is commonly produced. In the food industry, O/W nanoemulsions are desired to protect, increase the water-dispersibility, and deliver labile nutrients into food products. Emulsions are prepared using low (10^3^–10^5^ W Kg^−1^) or high-energy (10^8^–10^10^ W Kg^−1^) methods^3^. Among them, high-pressure homogenizer (HPH) as high-energy method, is the most frequent device to form nanoemulsions, allowing a large-scale production. For the target-compound to be dispersed into the aqueous phase, the reduction of the interfacial tension is crucial during the formulation process. Through the addition of sole surfactant or its combination with co-surfactant, lower the interfacial tension <3–10 mN/m^4^ is highly desirable, facilitating the breakdown process of the oil fraction in countless tiny droplets. Consequently, a high surface area is expected to be achieved (~70–330 m^2^/g)^2^, which can be convenient and useful to optimize the bioavailability of some target nutrients during digestion or alternatively, as an antimicrobial preservative.

Emulsions are affected by the rheological properties of the oil phase, which can directly influence its final application. Viscosity, density, and turbidity can affect drastically the sensorial perception or the background color of the food products. In turn, these modifications alter overwhelmingly the consumers’ purchase behavior^4^. Beyond this, emulsions are usually added to a complex food matrix, being susceptible to different environmental stress. This may occur due to the solution’s conditions (e.g., pH or salt concentration), storage-temperature, or time-spend on the shelf. Chemical degradation is also important to be assessed. Oxidation or volatilization rate should be tracked depending on the characteristics of the active ingredient. As a kinetic stable system, i.e., given enough time, emulsions are prone to breakdown due to different instability mechanisms^5^.The rate of the breakdown process depends on the surrounding environment that the colloidal system is exposed. Therefore, physicochemical studies that simulate these circumstances imposed by the food-process are very recommended^6^. However, mainly aiming at commercial application, nanoemulsions can be structured to hold robust metastability varying from months to years^3,7^

α-Terpineol (α-TOH) is classified as a tertiary alcohol monoterpenoid. Due to its floral, lilac-type, and sweet odor, α-TOH is one of the commonest ingredients used in the food industry as a flavoring agent, among other extensive applications^8^. As a result, its annually worldwide demand is ~330 tons^9^. Besides this, recent studies have been highlighting some novel biological functionalities for this monoterpenoid^10,11^, such as antitumor^12^; antidepressant^13^; anticonvulsant^14^; anti-inflammatory^15–17^, antidiarrheal^18^; antibacterial^19^ and antifungal^20^. α-TOH is recognized as a thermolabile and volatile molecule due to its low-molecular-weight (<300 g mol^−1^)^21^, as well as is moisture- and air-sensitive. Nonetheless, the trapping of this versatile monoterpenoid into O/W nanoemulsion as a sole active ingredient has not been fully described in scientific literature.

Despite of all advantages offered by the nanostructured systems, there are some drawbacks related to analysis techniques employed for their robust evaluation, which demands novel approaches. As highlighted by de Matos, Lucca & Koester (2019)^1^, a lot of emphasis is given to the physical stability studies of nanoemulsions loaded with flavor oils. However, in most of the studies (*c.a*. 60%) reported in the literature, the monitoring of the chemical degradation is frequently ignored. This omission can lead to a misrepresentative conclusion, mainly when volatile molecules are nanoemulsified as the active ingredient and its volatilization can result in the functionalization losses of the nanocolloidal system. Likewise, when the chemical degradation is investigated, indirect quantifications, mainly made by spectrophotometry are often used (*c.a*. 50% of studies); when gas chromatography analysis could be the most appropriate technique employed for direct quantification^1^. Another point that also worth investigation comprises the elucidation of whether the use of high-pressure as a high-energy formulation technique leads to the degradation of sensitive ingredients, like flavor oils, during the formulation of nanoemulsions^22^.

Hence, guided by the hypothesis that due to its own amphiphilicity as monoterpenoid alcohol, α-TOH may display co-surfactant activity, its interfacial ability was primarily investigated; followed by its oil-in-water nanoemulsion formulation (α-TOH-NE). Afterwards, driven by the second hypothesis that the stabilization of sensitive ingredient dispersed into the oil-in-water nano-colloidal system can support the extension of its physical and chemical stability, a defined formulation of α-TOH-NE was tracked for 410-days having the mean droplet diameter, turbidity, and marker content of α-TOH as the main parameters for monitoring overtime. Additional investigations were also assessed, like rheological properties of the α-TOH-NE, freeze-thaw cycle, pH, and salt concentration tests. Therefore, this study elucidates the features and stabilization of α-TOH into the nano-delivery system as a pioneering study and may its findings will be broadened beyond the food science field.

## Results and discussion

### Effect of α-TOH fractions on the interfacial tension (IFT) profile in absence of surfactant

**Fig. 1:**
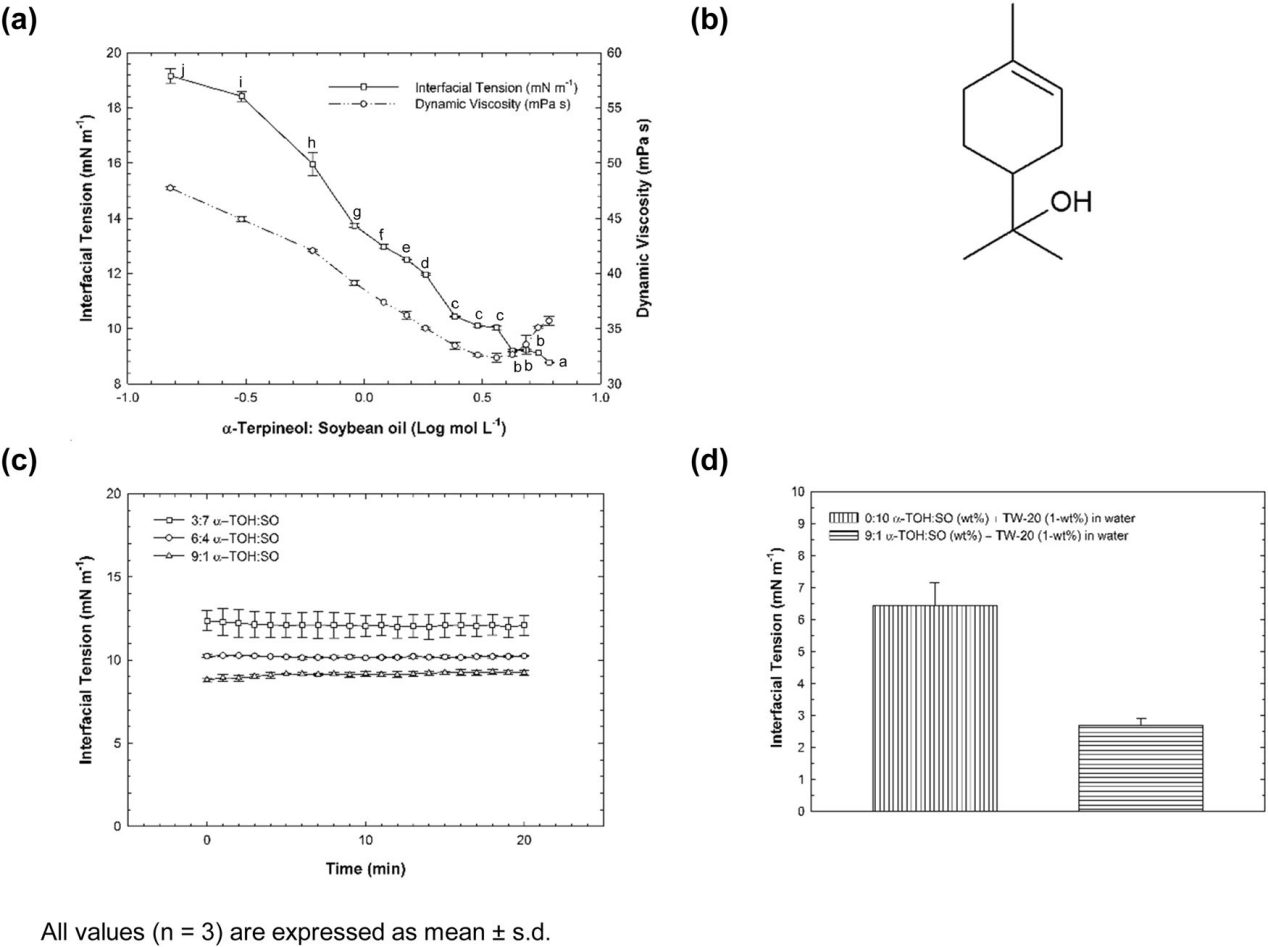
Plots exhibiting the ability of α-terpineol to low the interfacial tension on the oil-water interface. **(a**) Static interfacial tension (in absence of Tween^®^20, TW-20) for α-terpineol (α-TOH) blended with different ratios of soybean oil (SO) and its correspondent dynamic viscosity. Values accompanied by different lowercase letters (**a**–**j**) indicate significant differences (Duncan Multiple Range Test; *p* < 0.05). (**b)** Molecular structure of α-TOH (C_10_H_18_O; M_w_ = 154.25 g mol^−1^) showing its amphiphilic structure due to the polar and non-polar region. **(c)** Dynamic interfacial tension (in absence of TW-20) for some ratios of α-TOH: SO (3:7; 6:4; 9:1); (**d**) Static interfacial tension (in presence of TW-20) for α-TOH: SO (0:10; 9:1).

For different ratios of α-TOH: SO, the static IFT values are displayed in Fig. 1(a). The IFT of pure soybean oil (SO) (e.g., 0:10 α-TOH: SO) against water was 26.48 ± 3.19 mN m^−1^, a similar result as reported in the literature (y_ow_ = 26 ± 0.30 mN m^−1^)^23^. A sharp decrease from 19.15 ± 0.27 to 10.11 ± 0.01 mN m^−1^ was noticed for the nine lowest concentrations of α-TOH blended with SO. After this abrupted reduction, the IFT presented only a slight variation even the amount of α-TOH: SO has been increased until the sole presence of α-TOH (e.g., 10:0 α-TOH: SO). This abrupt reduction followed by a *plateau* was analogous to a critical micelle concentration (CMC) silhouette. These results indicated that α-TOH shows interfacial-activity, in agreement with previous interfacial properties reported for other flavors and essential oils by Turina, Nolan, Zygadlo, Perillo (2006) and Arneodo, Baszkin, Benoit, Fellous, Thies (1988)^24,25^. α-TOH (C_10_H_18_O; M_w_: 154.25 g mol^−1^) is sparingly soluble in water, exhibiting a partition coefficient (log K_ow_) and water solubility^26^ estimated as 2.6 and 2.4 g L^−1^, respectively. This value is 82 times higher than the water solubility of limonene (C_10_H_16_; M_w_: 136.24 g mol^−1^)^27^, its non-hydroxylated counterpart. The great water solubility presented by α-TOH is probably associated with the polar region of its molecule (Fig. 1(b)) due to the hydrogen bond donor –O^δ-^–H, which can form dipole-dipole interactions with water. In turn, its ring structure is a non-polar region. Therefore, like for other oxygenated monoterpenoids^26^, α-TOH presents an amphiphilic character, impacting directly in its ability to lower the IFT between two immiscible liquids.

Thus, from the sloped depicted in Fig. 1(a) for the lowest nine concentrations, the parameters that confirms the interfacial ability on the liquid-liquid interface for α-TOH are displayed on Table 1. With a CMC estimated as 0.48 mol L^−1^, we could demonstrate that the thermodynamic process of α-TOH to assembly on the liquid-liquid interface is a spontaneous process considering its negative value found for the Gibbs free energy (ΔG^°^_m_ = −1.81 KJ mol^−1^). The area estimated for the polar head group of α-TOH molecule (A_min_ = 20.29 Å^2^) in this study was in accordance with the topological polar surface area (TPSA, 20.2 Å^2^) reported for this compound in *PubChem*^©^ (based on computational calculation). When compared to the polar head group area of other surfactants—such as 60 Å^2^, 72 Å^2^ and 83 Å^2^ determined for Tween-20 (M_w_: 1225 g mol^−1^)^28^, Tween-80 (M_w_: 1310 g mol^−1^)^29^ and Quillaja saponin (M_w_: 1650 g mol^−1^)^30^, respectively – the polar head group found for α-TOH was relatively small. However, this is consistent with the definition of co-surfactants, i.e., surface-active molecules characterized by small polar head groups, which are usually observed in n-alcohols and alkyl carboxylic acids (C4-C10)^31^. The critical role played by the co-surfactant is mainly due to the ability to change the optimum curvature of the interfacial film. This alteration confers a better allocation and area covered by the surfactant molecules to the oil droplets, increasing their packing density, improving the formation and stability of the micelles^31–33^. In this sense, a larger value for *Γ*_*max*_ or reduced values for A_min_ is a signal of packing densities^34^ of surface-active molecules at the liquid-liquid interface. However, despite the CMC estimated and reported here for α-TOH, as well as the surface-active ability showed by some flavor oils, these molecules are not able to form and stabilize micelles by themselves owing to their small polar head group.

**Table 1:** Interfacial and thermodynamic property determined for α-terpineol (α-TOH) on the liquid-liquid interface in this study.

Parameter	Value
CMC (mol L^−1^)	0.48
${\upgamma}_{{{{\mathrm{CMC}}}}}$ (mN m^−1^)	10.11
Δ*G*°_m_ (KJ mol^−1^)	−1.81
*A*_*min*_ (Å^2^)	20.29
*Γ*_*max*_ (mol m^−2^)	8.19 × 10^−6^

For the dynamic IFT data (Fig. 1(c)) a negligible variation occurred over time (20 min) for all α-TOH: SO ratios (3:7; 6:4; 9:1) evaluated. This unchanged values confirm that α-TOH can quickly assemble on the interface, a similar behavior also observed for small molecule surfactants^35^.

### Effect of α-TOH on the IFT in presence of surfactant

According to Fig. 1(d), α-TOH contributed to reduce the IFT greatly when Tween^®^20 (TW-20) was into the aqueous phase. For the blending containing 9:1 α-TOH: SO against the 1-wt% TW-20 solution, the IFT was 2.73 ± 0.21 mN m^−1^. On the other hand, IFT was 6.45 ± 0.70 mN m^−1^ for pure SO (i.e., 0:10 α-TOH: SO) against 1-wt% TW-20 solution. Beyond water, due to its hydrophilic-lipophilic balance (HLB = 16.7), TW-20 also exhibits solubility in other polar solvents (i.e., alcohol, and ethyl acetate)^36^, indicating possibly its miscibility with α-TOH as an alcohol molecule. Thus, may this interaction lead to a synergic effect between α-TOH and TW-20 to lower the IFT, where α-TOH act as a co-surfactant. Other monoterpenoid alcohols like α-TOH have also been considered to act as co-surfactant, such as geraniol (GeOH) (C_10_H_18_O; M_w_ = 154.25 g mol^−1^). In studies reported by Stubenrauch, Paeplow, Findenegg (1997)^32^, it was demonstrated that GeOH was organized in a regular manner within the interfacial film. In this case, two molecules of GeOH were found for every five molecules of β-octyl monoglucoside as a surfactant. Therefore, we suggest that α-TOH and TW-20, when combined in the same colloidal system, may be organized in an analogous way.

### Formulation of oil-in-water nanoemulsion using different fractions of α-TOH

The nanoemulsions (NEs) loaded with different ratios of α-TOH: SO (10-wt% of the oil phase) were successfully formulated and the results are presented in Table 2. For all freshly prepared NEs (0αTOH-NE, 3αTOH-NE, 6αTOH-NE, 9αTOH-NE), our results indicated that pH (~7) or conductivity (~1.2 mS/cm) were not affected by the composition of the oil phase for all colloidal systems (no statistical difference found for both values, *p* < 0.05). Moreover, as reported in the literature, the mean particle diameter (MDD) of all NEs fall in the range as expected for nanoemulsion size: 20 < *d* < 200 nm and low polydispersity index <10%^2,4^. By visual inspection, all NEs exhibited a slightly turbid appearance like milk-color. This observation confirms that the MDD found for all these NEs was higher or fairly similar to the wavelength of the visible light (100 nm <*d* < 200 nm, see Table 2), consequently, the incident light was intensely scattered, leading to an opaque appearance perception^2–4^.

**Table 2:** Nanoemulsions prepared using different α-terpineol: soybean oil ratios as 10-wt% an oil phase and its values^1^ for different parameters: ζ-potential, conductivity, and particle size characterization for 0 and 90-days.

0-day					90-day	
*Nanoemulsion*	*ζ-potential (mV)*	*Conductivity (mS/cm)*	*MDD* ^*2*^ *(nm)*	*PdI* (%)	*MDD (nm)*	*PdI* ^*3*^ (%)
0αTOH-NE	−14.2 ± 0.5^a^	1.1 ± 0.04^a^	173.3 ± 0.7^d^	7.7 ± 1.1^a^	173.2 ± 0.1^b^	9.80 ± 2.5^b^
3αTOH-NE	−13.4 ± 0.6^a,b^	1.2 ± 0.04^a^	155.3 ± 1.1^a^	4.6 ± 1.1^a^	157.4 ± 0.3^a^	6.80 ± 2.1^a,b^
6αTOH-NE	−12.8 ± 0.3^b,c^	1.2 ± 0.04^a^	168.0 ± 0.9^c^	5.3 ± 2.5^a^	176.0 ± 1.2^c^	4.20 ± 0.6^a^
9αTOH-NE	−11.8 ± 0.5^c^	1.2 ± 0.04^a^	164.7 ± 2.3^b^	7.5 ± 1.2^a^	173.6 ± 0.3^b^	6.97 ± 0.92^a,b^

^1^Values (*n* = 3; mean ± s.d.) accompanied by different lowercase letters (a–d) indicate significant differences among the samples (Duncan Multiple Range Test; *p* < 0.05). ^**2**^ MDD: mean droplet diameter; ^**3**^ PdI: Polydispersity Index.

### Droplet charge

For ζ-potential (Table 2), once the fraction of α-TOH into the NE increased, the ζ-potential reduced roughly 20% between the lowest (0αTOH-NE) and highest concentration (9αTOH-NE) of α-TOH-loaded NEs. These results indicated that ζ-potential was affected by the volume fraction of α-TOH in the lipid phase, where a statistical difference (*p* < 0.05) was found among the samples for this parameter. Having α-TOH as a non-ionizable polar molecule and TW-20 as a nonionic surfactant^29^, the reduction perceived in the absolute value for ζ-potential was expected. This additional droplet charge found for the 0αTOH-NE may be attributed to lecithin which holds a negative charge and may be found frequently in soybean oil as a residue^37^. ζ-potential is one of the parameters able to predict the stability of the colloidal system through the magnitude of particles’ charge^37^. As a reference, ζ > |30| can confer great stability to the colloidal system^38^. Although the magnitude of ζ-potential values found in this study was < |30|, the formulated NEs were stable since other factors can affect the stability of colloidal systems, such as the addition of emulsifiers^39^. In this case, TW-20 may have been able to prevent the droplet aggregation by steric repulsion due to its large head group (hydrophilic portion)^40^.

### Stability for 90^th^ day

For the stability up to 90^th^ day, control nanoemulsion (0αTOH-NE) showed a negligible change in the MDD over time, where no statistical difference *p* < 0.05 was found when the MDD between the 0th and 90^th^ day were compared (Table 2). The low water solubility of soybean oil as long-chain triglycerides might explain this phenomenon delaying the Ostwald ripening (OR)^41^. For nanoemulsions prepared using different ratios of α-TOH: SO (3αTOH-NE, 6αTOH-NE, 9αTOH-NE) results revealed that the MDD increased as time passed, where a statistical difference (*p* < 0.05) was found for MDD on 0th and 90th day and between the samples on 90^th^ day. This may be linked to OR considering the high solubility of α-TOH in the aqueous phase. However, even with the rise of MDD, for both parameters, i.e., growth rate and polydispersity index, a minor variation (<10%) was noticed from 0 to 90^th^ day. Therefore, considering this stability, 9αTOH-NE was considered for further physicochemical stability studies as it will be detailed in the next sections.

We hypothesize that this high-load flavor oil NE (~90 mg mL^−1^) of α-TOH showed this appreciable long-term physical stability due to its co-surfactant activity (as discussed previously), aiding greatly itself in its own encapsulation. The synergistic effect between TW-20 and α-TOH to lower the interfacial tension (<3 mN m^−1^) possibly had a great impact on the effectiveness to break down the lipid fraction into tiny oil droplets during the homogenization process, leading to robust metastability of its nanoemulsion along with time.

Moreover, the blending formed by α-TOH and SO may play a key role to delay the OR rate (ω). Different studies indicate that the proportion between “ripening inhibitors” and some flavor oils are specific for every NE-system, such as thyme oil: corn or MCT oil in the ratio of 4:6 or 5:5 (wt.%)^42^; orange oil: MCT oil in the proportion of 5:5 (wt.%)^43^; and clove oil: MCT or corn oil blended as 2.5:7.5 or 5:5 (wt.%)^44^, respectively. Furthermore, as reported by Suriyarak & Weiss (2014)^45^, the equilibrium between antimicrobial activity and physical stability should be found for the NEs-loaded with essential oils. Therefore, in our system, the ratio of α-TOH: SO as 9:1 is confirmed optimal for the oil phase composition. The high concentration of α-TOH could be desirable for an antimicrobial application for 9αTOH-NE, while reducing the concentration of ripening inhibitor. As demonstrated by Chang, McLandsborough, McClements (2014)^42^, the antimicrobial efficacy of thymol oil-loaded NE was positively affected when the concentration of active-ingredient was increased, while the amount of ripening retardant was reduced.

### Effect of storage-temperature on the dynamic viscosity and density measurements

Density and dynamic viscosity measurements for 9αTOH-NE are displayed in Table 3. Similarly, to pure water and other fluids, the dynamic viscosity and density of 9αTOH-NE indicated a thermal dependency (statistical difference *p* < 0.05), increasing once the temperature was reduced. Because of the high viscosity and less density character of the oil phase (α-TOH; SO), the dynamic viscosity and density were always higher and lower when compared to pure water, respectively. α-TOH is prone to crystallization under its melting point (<35 °C)^8^, thus possibly influenced the high dynamic viscosity value of the 9αTOH-NE at a lower temperature.

**Table 3:** Dynamic viscosity and density values ^1^ found for 9αTOH-NE at 5, 25, and 50 °C compared to pure water.

Temperature (°C)	Dynamic viscosity (mPa s)		Density (g cm^−3^)	
	*9αTOH-NE*	*Water* ^2^	*9αTOH-NE*	*Water* ^2^
*5*	2.40 ± 0.10^c^	1.52	0.997 ± 0.17^c^	1.000
*25*	1.33 ± 0.13^b^	0.89	0.945 ± 0.11^b^	0.997
*50*	0.89 ± 0.35^a^	0.54	0.934 ± 0.33^a^	0.988

^1^Values (*n* = 3; mean ± s.d.) accompanied by different lowercase letters (a–c) indicate significant differences (Duncan Multiple Range Test; *p* < 0.05). ^2^https://wiki.anton-paar.com/en/water/

Rheological properties of NEs are important to the texture of foods and can impact the sensorial perception of consumers. For instance, for drinks with low viscosity, a change in their overall viscosity due to addition of NE is undesired^46^.

### Environmental stress: effect of storage-temperature overtime (410^th^ day) on turbidity

**Fig. 2:**
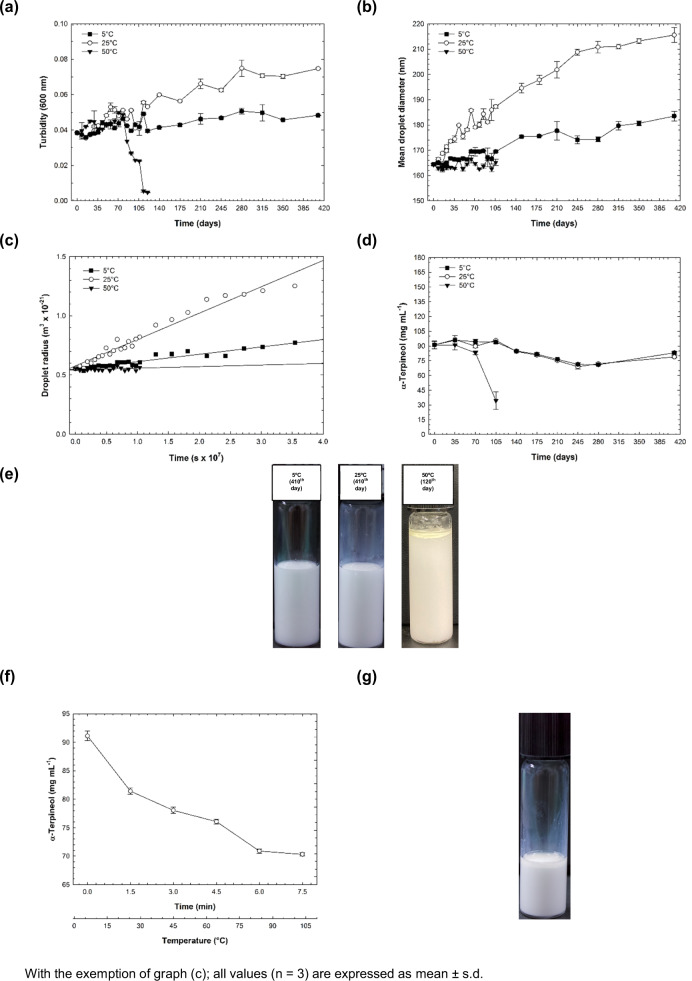
Plots for stability studies of 9αTOH-NE stored at 5, 25, and 50 °C. (**a**) Turbidity at 600 nm for 9αTOH-NE watery 200-fold; (**b**) Mean droplet diameter (nm) *versus* time (410-days); (**c**) The cube of the droplet radius (r^3^) against time (s); (**d**) Retention of nanoemulsified α-TOH (mg mL^−1^) into 9αTOH-NE as time passed. Values accompanied by different lowercase letters (a-j) indicate significant differences (Duncan Multiple Range Test; *p* < 0.05). (**e**) Photographs of the 9αTOH-NEs. Not noticed by the picture, but for 9αTOH-NE at 50 °C a phase separation was also observed in the middle of the glass vial additionally to the oil accumulation on the top. (**f**) Simulation of cooking process and their impact on the retention of nanoemulsified α-TOH (mg mL^−1^) as a function of time (min) and temperature (°C); (**g**) Photograph of the 9αTOH-NE submitted to the cook process simulation (after 5 min at 105 °C) where an oiling off can be observed. Considering the high content of water into the 9αTOH-NE, the final volume was reduced *c.a*. 5-fold compared to the initial volume.

Turbidity observed for long-term storage (410^th^ day) of 9αTOH-NE incubated at 5, 25, and 50 °C is shown in Fig. 2(a). For freshly prepared 9αTOH-NE the turbidity was ~0.038 (0^th^ day). Then, up to 410^th^ day, the turbidity varied ~0.048 (9αTOH-NE at 5 °C) and ~0.075 (9αTOH-NE at 25 °C). For 9αTOH-NE at 5 and 25 °C, the increasing on turbidity displayed a thermal-dependence, where the increment-rate for this parameter for 9αTOH-NE at 25 °C was higher (97%) than at 5 °C (26%). This dependency to the temperature observed for the turbidity was confirmed by statistical difference (*p* < 0.05) found when the whole data set were compared individually between the 0^th^ and 410^th^ day.

For 9αTOH-NE at 50 °C, the turbidity increased continuously up to ~0.050 (78th day). Then, a sharp decrease was observed from ~0.034 (85th day) to 0.005 (120th day). This drastically changing in the turbidity for 9αTOH-NE at 50 °C may be due to the accumulation of the lipid fraction on the top of the tube test. Through visual observation, an oil accretion began to be perceived from the 85th day, culminating in the emulsion breakdown at 50 °C after 120th day. Droplet collisions that lead to the destabilization mechanism are more frequent when the thermal energy is higher. Therefore, oiling-off contributed to lowering the overall turbidity of 9αTOH-NE at 50 °C. These results differ from the study reported by Li & Lu (2016)^47^ for limonene-loaded nanoemulsion (LIM-NE) produced by ultrasound and monitored for 98^th^ day. In this case, for LIM-NE kept at 5 and 25 °C no alterations were found, while for LIM-NE at 50 °C a constant and high-rate on the growth of turbidity was demonstrated. The difference found between these two studies may be related to the oil phase composition and the means used for nanoemulsion preparation.

### Environmental stress: effect of storage-temperature overtime (410^th^ day) on mean droplet diameter (MDD)

The MDD monitored for 410th day for 9αTOH-NE at 5, 25, and 50 °C is displayed in Fig. 2(b). For freshly prepared 9αTOH-NE, the droplet size found was ~164 nm (0th day). Then, up to 410^th^ the size increased up to ~ 184 nm (9αTOH-NE at 5 °C) and ~216 nm (and 9αTOH-NE at 25 °C), where a statistical difference (p < 0.05) was found when the MDD values of the entire data were compared between the 0 and 410^th^ day. At 5 °C, a time-growth dependence started to be more pronounced from 120-day, while for 9αTOH-NE at 25 °C this tendency was noticed since the 0-day. Through visual observation, no alterations were observed for 9αTOH-NE at 5 and 25 °C (Fig. 2(e)), despite the temporal growth of their droplet size. Oppositely, for 9αTOH-NE at 50 °C, the droplet size did not change over time (no statistical difference *p* < 0.05 was found between the 0 and 120^th^ day), with an average of ~165 nm up to 120th day. However, an oiling off started to be clear perceived by naked eyes since the 85th day.

According to the LSW (Lifshitz-Slezov-Wagner) theory, Ostwald ripening (OR) was proposed as one of the irreversible destabilizing mechanisms that lead to NEs breakdown overtime^48^. As highlighted by Nazarzadeh, Anthonypillai, Sajjadi (2013)^49^, OR is validated by the LSW model if some specific conditions are satisfied: (i) highly diluted emulsions; (ii) over time, successive ups and downs of the droplet size is observed, depicted by a tooth-saw-like behavior; (iii) an increase linear correlation between the cube of the droplet radius (r^3^) of the NE against time (s) is found. In this study, the first condition is satisfied, once 9αTOH-NE is highly diluted (10:90 oil fraction: continuous phase). Second, in (Fig. 2(b)), for 9αTOH-NE at 5 and 25 °C, a tooth-saw-like behavior evolution of the droplet size growth as time passed was also noticed. Thereby, a satisfactory regression coefficient (R; *p* < 0.05) for the cube of the droplet radius *versus* time was found for 9αTOH-NE at 5 and 25 °C. Thus, considering the first term of the Eq. (1), the OR rate (ω) was determined from the slope depicted in Fig. 2(c). The results revealed a ω value of 6.22 × 10^−30^ m^3^ s^−1^ (*R* = 0.9514) for 9αTOH-NE kept at 5 °C from 0 to 410th day; while this value for 9αTOH-NE kept at 25 °C was initially (from 0 to 280^th^ day) equal to 26.65 × 10^−30^ m^3^ s^−1^ (*R* = 0.9884), then dropped to 8.64 × 10^−30^ m^3^ s^−1^ (from 315 to 410th day, *R* = 0.9967), indicating the kinetic of the ripening process began to decrease over time. Similar observation between the reduction of the droplet growth leading to a slower kinetic of ω is also reported by Li & Chiang (2012); Du, Wang, Tai, Wang, Liu (2016); Verma, Kumar, Gokhale, Burgess (2011)^50–52^, which may be related to the approaching of the maximum saturation solubility of the oil phase into the water phase.

The difference found for ω at 5 and 25 °C is may due to the thermal dependency of the interfacial tension (γ) as well as the solubility (C ∞ ) and diffusivity (D) of the oil phase, which increase once the temperature also rises, as stated by Gupta, Eral, Hatton, Doyle (2016) and Verma, Kumar, Gokhale, Burgess (2011)^3,52^.

1 $$\omega = \frac{{dr}^3}{dt} = \frac{{8{{{{C}}}}\infty \gamma {{{\rm{VmD}}}}}}{{9{{{{\uprho }}}}{{{\rm{RT}}}}}}$$

Where, *ω* = Ostwald ripening rate; r = mean droplet radius (nm), t = time (s), C_∞_= bulk solubility of oil fraction in water-phase (mol m^−3^), γ = interfacial tension (N m^−1^), V_m_ = molar volume of oil phase (m mol^−1^), D = diffusion coefficient of the lipid fraction in the water-phase (m^2^ s^−1^), ρ = density of the oil (Kg m^−3^), R = gas constant (8.314 J mol^−1^ K^−1^), T = absolute temperature (K).

In contrast, for 9αTOH-NE at 50 °C, despite the whole alteration displayed on the turbidity for this nanocolloidal system at the same temperature, no linear correlation was encountered for the temporal evolution of the droplet radius in 9αTOH-NE at 50 °C (Fig. 2(c)). Therefore, probably for 9αTOH-NE at 50 °C, the dominant growth mechanism was different if compared to 9αTOH-NE at 5 and 25 °C. Flocculation and gravitational separation may be the dominating demulsification process of 9αTOH-NE at 50 °C. Flocculation consists of two or more droplets combined, but their individuality is kept. Since sample dilution is performed before droplet size measurement, floccules desegregation might take place. This desegregation could explain the absence of differences in particle size for 9αTOH-NE at 50 °C during the monitoring period. This hypothesis was also proposed by Yang, Leser, Sher, McClements (2013)^29^, who observed no alterations in the droplet size despite an oiling-off noticed in their nanocolloidal system. Gravitational separation may also occur simultaneously in the 9αTOH-NE at 50 °C due to the density difference between the oil fractions and the aqueous phase.

The difference found in our results indicates that the incubation-temperature might influence the rate and which destabilizing mechanism will dominate for the NE-breakdown overtime. As a result of the thermal influence, a shift in the mass transport kinetics of the lipid and surfactant fraction occurs, affecting the arrangement on droplet oils’ interface^47^. Thus, understanding the temperature-response behavior of NEs is fundamental, once plays a key role in determining the ideal storage conditions, directly impacting the shelf-life of the product in the real market condition.

### Environmental stress: effect of storage-temperature overtime (410th day) on the retention of the α-TOH nanoemulsified

First, the non-isothermal Kovats retention index (RI) for α-TOH was determined. The RI found was ~1706 iu (index unit), falling in the range reported for α-TOH in PEG-column (~1694 ± 19.4 iu)^53^. Next, the sensitivity of the method used for α-TOH quantification was calculated through a calibration curve (Supplementary Fig. 1(b)). Values equal to 1.34 and 4.06 mg mL^−1^ were found as the limit of detection (LOD) and quantification (LOQ), respectively. Once these conditions were fixed, the quantification of α-TOH retained in 9αTOH-NE at 5, 25, and 50 °C was monitored for 410-days. The results are shown in Fig. 2(d).

For newly prepared 9αTOH-NE (0^th^ day) the concentration found for α-TOH was ~90 mg mL^−1^. Surprisingly, this it was the exact amount of α-TOH initially added to the oil phase aimed for its nanoemulsification. High-pressure homogenizer (HPH) is known as a high-energy method for NEs preparation. This means that great friction heating happens throughout the process. As a thermolabile molecule, it was expected that a considerable amount of α-TOH could be lost during the 9αTOH-NE preparation using HPH. However, the encapsulation efficiency was *c.a*.100%. This result is aligned with conclusions reported by Håkansson (2019)^22^, where it was demonstrated that independently of the pressure, the residence time (3–40 ms) on the HPH valve is too short to lead to a thermal-degradation of the labile molecules. Likewise, as showed by Cecchini et al. (2021)^54^, negligible alteration (<1%) of the relative composition/abundance was found when the pure and the nanoemulsified essential oil of *M. verticillata* was evaluated by chromatography analysis prior to and after the emulsification procedure.

The results regarding the retention of α-TOH into 9αTOH-NE overtime showed a strong response behavior related to the preservation of the physical integrity of the NE. For 9αTOH-NE incubated at 5, 25, and 50 °C, no statistical difference (*p* < 0.05) was found among the samples for the concentration of α-TOH (*c.a*. 90 mg mL^−1^) up to 70^th^ day. On contrary, for 9αTOH-NE at 5 and 25 °C, despite the clear difference found on the droplet size growth as discussed previously, the chemical stability regarding the mark-content of α-TOH indicated a non-thermal dependence up to 410th day between both nano-colloidal system. As noticed in the Fig. 2(d), the amount of α-TOH retained into the 9αTOH-NE at 5 and 25 °C showed a similar trend and silhouette for both storage-temperatures. Thus, on 410^th^ day, the α-TOH content into the nanoemulsion after incubation at 5 and 25 °C was *c.a*. to 92 and 88% of the α-TOH amount initially encapsulated (0th day) respectively. These results are like the values reported for other terpene compounds in other studies. For instance, Ghaderi, Moghmi, Aliahmadi, McClements (2017)^55^ found that *c.a*. 96 and 93% of thymol-oil content remained into two different formulation of O/W nanoemulsion after incubation for 180 days (storage temperature was not mentioned). Similarly, silica nanoparticles (SNPs) loaded with linalool and α-pinene contributed to the life-extension of both terpenes when incubated at 45 °C for 180 days^56^. Likewise, Tubtimsri et al. (2021) showed that the nanoemulsified spearmint oil presented a carvone (main compound in this essential oil) retention over 90% when kept at 40 °C for 180 days^57^.

For 9αTOH-NE at 50 °C, impacted by the oiling off which started to take place from 85th day, the loss of α-TOH showed a sharp decrease in a short period of time (25 days), where *c.a*. 40% of the nanoemulsified α-TOH was found into the nanocolloidal system on 105th day. Therefore, we demonstrated how critical is the maintenance of the overall integrity of NE for the effective retention of active ingredients encapsulated into NEs and its tight dependency on the storage-temperature.

### Environmental stress: simulation of cooking process

The cooking simulation conditions of the 9αTOH-NE indicated a continuous loss of α-TOH retained into the nanoemulsion, since the temperature was constantly increasing along with time (Fig. 2(f)). Thus, after 7.5 min of cooking process, the amount of α-TOH retained into the NE was nearly to 77% of the original amount added for the NE formulation, with a loss-rate estimated as 2.7 mg mL^−1^ min^−1^ during the cooking process. After 5 min cooking at 105 °C, the mark-content found for α-TOH was *c.a*. to 27% of the initial concentration, showing a loss rate estimated as 9.4 mg mL^−1^ min^−1^. At the end of the cooking process, an evident layer of oiling off was noticed by naked eyes in the cooked sample, indicating that a demulsification process took place into 9αTOH-NE (Fig. 2(g)).

The cloud point (CP) reported for TW-20 is 76 °C^58^. At or beyond the temperature of CP, dehydration of the head group of the non-ionic surfactant molecules—such as TW-20—start to occur. As a result, the optimum surfactant monolayer curvature is altered, turning the NE more susceptible to coalescence^59^, leading to the oiling off accumulation. Due to the demulsification process and the displacement of the surfactant molecules which covers the oil droplet, the migration of water to the interface of these droplets causes cracks, leading to the release and exposition of the entrapped flavor to the harsh external environmental conditions. The increasing of the thermal energy also increases the diffusion rate of the lipid fraction to the aqueous phase as well as the collision frequency of these oil droplets^47^. Thereby, in practical terms, during the food processing, is important to keep the nano colloidal system apart from the droplet coalescence zone caused by the augmentation of the temperature over the CP for that NEs which are stabilized by nonionic surfactants^59^.

### Environmental stress: freeze-thawing

The impact of a freeze-thawing on the stability of 9αTOH-NE nanoemulsion was assessed. Complete phase separation of the NE was noticed after a 1-cycle of freeze-thawing. The mean droplet size was ~243 nm, showing a high polydispersity index ~46%. The prominent droplet aggregation that culminated on 9αTOH-NE breakdown was probably due to the coalescence phenomena. As mentioned by McClements (2004)^60^, once the colloidal system is frozen, water crystallizes and confines the oil droplets in the remaining liquid space. This confining of the lipid fraction increases droplet-droplet interactions, causing the interfacial film rupture, leading to the emergence of larger droplets (coalescence). Water crystallization also results in the dehydration of the surfactant molecules present at the interface, causing its removal from the interfacial layer. Freeze-thaw stability can also be influenced by the nature of the emulsifier molecule. Low molecular weight (LMW) surfactants molecules like TW-20, show high sensitivity for freeze and thawing procedures since they can cover the oil droplets just with a thin interfacial layer, which makes fat droplets more vulnerable to film rupture as the key-mechanism for coalescence^61^.

### Environmental stress: solution condition tests

**Fig. 3:**
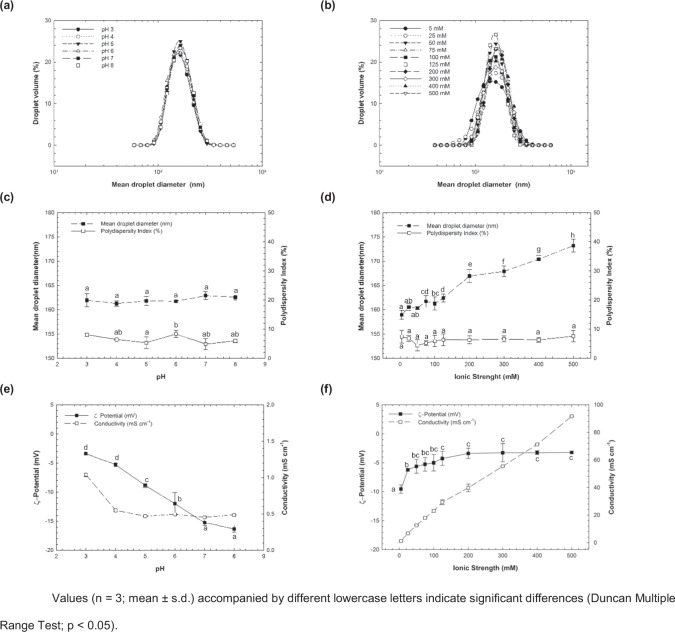
Solution condition tests applied to the physical-stability studies of 9αTOH-NE at 25 °C. (**a**) and (**b**) Particle size distribution for pH (3–8) and salt concentration assessments; (**c**) and (**d**) Mean droplet diameter and polydispersity index for pH and salt concentration analyses; (**e**) and (**f**) ζ-potential and conductivity found for pH and ionic concentration protocols.

The influence of the different solution conditions, like pH and salt concentration, on the stability of 9αTOH-NE was investigated (Fig. 3). The results indicate that the 9αTOH-NE was stable to droplet aggregation regardless of the pH (3–8) or salt concentration (5–500 mM) in the aqueous phase. This was confirmed by the superposed, monomodal particle size distribution curves and low polydispersity index (<10%) for both pH and salt concentration tests (Fig. 3(a)–(d)). For pH treatment, the mean droplet size showed a negligible alteration (no statistical difference was found, *p* < 0.05), varying from ~162 (pH 2) to 163 nm (pH 8) (Fig. 3(c)). On the other hand, for the salt concentration treatment, a variation marked by statistical difference (*p* < 0.05) in droplet size was observed from ~159 nm (5 mM) to ~173 nm (500 mM) (Fig. 3(d)). For droplet charge, changings were perceived along with the range of pH treatments applied (Fig. 3(e)), varying from ~|3| mV (pH 2) up to ~|16| mV (pH 8). For ionic strength treatment (Fig. 3(f)), the droplet charge variation accounted from ~|10| mV (5 mM) to ~|3| mV (500 mM). In both cases, a statistical difference (*p* < 0.05) was found for the droplet charge parameter.

Similar results are found in the literature regarding the physical stability of droplet oils covered by TW-20 as a surfactant. Studies made by Chen et al. (2018) and Taarj et al. (2018)^40,62^, showed that TW-20 was described as highly stable and relatively insensitive to the pH-range applied. The same applies to the stability presented by salt concentration treatment in a range of 0 to 500 mM, where no particle size alteration was perceived by the study conducted by Teo et al. (2016)^63^. As pointed out by Degner, Chung, Schlegel, Hutkins, McClements (2014)^61^, the insensitivity for the droplet aggregation of nonionic surfactant-coated oil droplets is guaranteed by the stearic stabilization instead of electrostatic repulsion^61^. This explains why no phase separation and small alterations were found for all pH or salt concentration-range tested for 9αTOH-NE at 25 °C.

## Conclusion

In this study, it was showed that α-TOH may act as a co-surfactant due to its ability to lower the interfacial tension on the oil-water interface in the absence or presence of surfactant into the aqueous solution. The long-term stability tracked from 0 to 410th day for 9αTOH-NE loaded with high-amount of α-TOH (90 mg mL^−1^), showed some key-findings: (i) the preparation of this nanocolloidal system by high-pressure homogenizer did not cause degradation to the initial amount of α-TOH added for its O/W nanoemulsion formulation; (ii) despite the high-susceptibility of α-TOH to degradation depending on temperature, moisture, and air exposition, the chemical stability of this sensitive bioactive ingredient was extended over than 1-year through its nanoemulsion formulation; (iii) the adapted gas chromatography analysis here proposed using a low amount of solvent and simple-preparation, was feasible to track the retention of α-TOH into the nanoemulsion overtime. Moreover, in a long term, the preservation of the physical stability integrity of this nanocolloidal system displayed a crucial role in the maintenance of the retention rate of the nanoemulsified α-TOH. Overall, the physical stability of 9αTOH-NE showed a thermal dependence for all the storage temperatures considered (5, 25, 50 °C); while for the chemical stability, the reduction amount of α-TOH into 9αTOH-NE kept at 5 and 25 °C showed a similar decaying-rate.

Thus, some additional investigations may be considered as an extension of the research here presented, such as: (i) although the usage of TW-20 is recognized as safe by several regulation compliances around the world, the replacement of TW-20 as a synthetic option by some natural one, such as crude extracts emulsifier obtained from agro-industrial waste products, plant- or biotechnology-based surfactants, should be considered as more eco-friendly and natural alternative; (ii) considering its co-surfactant activity, may the amount of the surfactant necessary to stabilize α-TOH-NE can be reduced, impacting on the final-cost of the formulation; (iii) analysis of the physicochemical stability of α-TOH-NE in a food-model aiming to simulate the real-market application; (iv) further studies focused solely on the gastrointestinal fate and the bioavailability of the α-TOH-NE as a crucial step to validate its commercialization to final consumers.

## Methods

### Chemicals & consumables

α-Terpineol (≥96% purity, natural); Polyoxyethylene(20) sorbitan monolaurate (Tween^®^20; HLB: 16.7) and alkane standard solution C_8_–C_20_ were acquired from Sigma-Aldrich (Tokyo, Japan). Ammonium sulfate ((NH_4_)_2_SO_4_); methanol (CH_3_OH); sodium azide (NaN_3_); sodium chloride (NaCl); sodium phosphate, monobasic (NaH_2_PO_4_ 2H_2_O); sodium phosphate, dibasic (Na_2_HPO_4_ 12H_2_O) and soybean oil were purchased from FUJIFILM Wako Pure Chemical Corporation (Osaka, Japan). All products here mentioned were reagent grade and used without any further purification. RephiQuick non-sterile syringe filter (PTFE 32 mm; 0.45 μm) was acquired from RephiLe Bioscience (Shangai, China). Ultrapure grade water (ASTM Type 1/0.055 μS cm^−1^/>18.2 MΩ.cm at 25˚C) was processed by Arium^®^ Comfort II (Sartorius AG, Göttingen) system.

### Interfacial tension (IFT) measurements

Liquid-liquid interfacial tension (IFT) of oil against water was acquired, using an inverted pendant drop method and determined by the Young-Laplace Equation calculated by FAMAS software (Kyowa Interface Science Co. Ltd, Saitama, Japan). Before the IFT measurements, the density values (showed in Supplementary Fig. 1(a)) for the oil phase were determined.

(i) *Effect of α-terpineol fractions into the oil phase in the absence of surfactant*—*static IFT:* to investigate the interfacial activity of α-terpineol, first, the static IFT of oil against water (in the absence of Tween^®^20) was acquired with an oil phase prepared by 14 different ratios (%v/v) of α-TOH: SO (0.25: 9.75; 0.50: 9.50; 1.0: 9.0; 1.5: 8.5; 2.0: 8.0; 2.5:7.5; 3.0: 7.0; 4.0: 6.0; 5.0: 5.0; 6.0: 4.0; 7.0: 3.0; 8.0: 2.0; 9.0: 1.0; 10.0: 0.0) as the protocol adapted from Kim and Burgess (2001)^64^. Based on the static profile of the static IFT using different ratios of α-TOH: SO blends and following the methodology from Chen et al. (2021) and Bergfreund, Siegenthaler, Lutz-Bueno, Fischer^33,34^, the follow parameters were determined for the pure α-TOH as follow: (*a*) the critical micelle concentration (CMC) determined from the breakpoint found between the concentration curve and the interfacial tension; (*b*) the Gibbs free energy (${\Delta}{{{{G}}}}_{{{\mathrm{m}}}}^{{{\mathrm{O}}}}$; KJ mol^−1^) (Eq. (2)); (*c*) the surface density of α-TOH at saturation (Ґ_max_; mol m^−2^) (Eq. (3)); (*d*) the minimum area occupied per head group of the α-TOH molecule (${{{{A}}}}_{{{{\mathrm{min}}}}};$ Å^2^) (Eq. (4)). Where R is the gas constant (8.314 mol^−1^ K^−1^); T is the absolute temperature (298.15 K); n is equal to 1 for non-ionic surfactant (or n = 2 for ionic surfactant); $\left( {\partial \gamma /\partial {{{{logC}}}}} \right)$ is the slope of the curve of the static interfacial tension (mN m^−1^) against logarithm (base-10) of α-TOH concentration (mol L^−1^); ${{{\rm{N}}}}_{{{\rm{A}}}}$ is the Avogadro constant (6.02 × 10^23^ molecules mol^−1^).
  2 $${\Delta}{{{{G}}}}_{{{\rm{m}}}}^{{{\rm{O}}}} = {{{{RT}}}}\ln {{{{CMC}}}}$$
  3 $$\gamma _{{{{\rm{max}}}}} = - \frac{1}{{2.303\,{{{\rm{n}}}}{{{{RT}}}}}}\left( {\frac{{\partial \gamma }}{{\partial {{{{{log}C}}}}}}} \right)$$
  4 $${{{{A}}}}_{{{{\rm{min}}}}} = \frac{1}{{\gamma _{{{{\rm{max}}}}}{{{{N}}}}_{{{{A}}}}}}$$
(ii) Effect of α-terpineol fractions into the oil phase in the absence of surfactant—dynamic *IFT:* the dynamic IFT (20 min) was recorded for three different ratios (%wt) of α-TOH: SO (3.0: 7.0; 6.0: 4.0; 9.0: 1.0).
(iii) Effect of α-terpineol in the oil phase on the IFT in presence of surfactant*:* to assess the IFT reduction when α-terpineol and TW-20 were combined in the same system, liquid-liquid IFT of oil against the aqueous system was acquired. The system was composed by α-terpineol: soybean oil (0:10) and 1-wt% TW-20 in water; α-terpineol: soybean oil (9:1) and 1-wt% TW-20 in water.

### Preparation of oil-in-water nanoemulsion

Coarse emulsions were formed by blending 10-wt% of the oil phase with 90-wt% of the aqueous phase. The aqueous phase consisted of 5 mM sodium phosphate buffer (pH 7.0), sodium azide (0.02-wt%), and 1-wt% TW-20 as a nonionic surfactant, fixing the surfactant: oil ratio (SOR) as 1:10. The oil and aqueous phases were mixed using a high-speed mixer (Polytron^®^, System PT-3100, Kinematica AG, Lucerne, Switzerland) operating at 7000 rpm for 5 min. Fine emulsions were then formed using high-pressure homogenization (HPH) to reduce the size of the liquid particles. Then, 100 g of coarse emulsions were processed in the homogenization unit for 3 passes (NanoVater, NV200, Yoshida Kikai, Nagoya, Japan) at 90 ± 2 MPa.

### Formulation of oil-in-water nanoemulsion containing different ratios of α-terpineol: soybean oil

To find the balance between the physical stability and maximize the encapsulation of the α-terpineol as active-ingredient, a preliminary experiment was conducted. Then, an O/W-NE loaded with α-terpineol was prepared by varying the composition of the 10-wt% of oil phase, which contained different ratios of α-TOH: SO as showed in Table 4. All these stocks O/W-NE was stored at 25˚C for 90 days. Periodically, samples were withdrawn for the mean droplet diameter measurements.

**Table 4:** The composition of the 10-wt.% of the oil phase O/W-NE using different ratios of α-terpineol: soybean oil.

Nanoemulsion label	Soybean oil (wt.%)	α-Terpineol (wt.%)	Concentration of α-Terpineol into O/W-NE (mg mL^−1^)
*0αTOH-NE*	10	0	0
*3αTOH-NE*	7	3	30
*6αTOH-NE*	4	6	60
*9αTOH-NE*	1	9	90

### Environmental stress tests and its effect on the physicochemical stability of 9αTOH-NE

The stability of the 9αTOH-NE was tested, following the protocol from McClements & Gumus (2016)^6^ and Wang, Doi, McClements (2019)^65^, with slight modification.

(i) *Effect of thermal processing combined with long-term stability:* 10 ml of the freshly prepared 9αTOH-NE (pH 7; 5 mM) was placed in glass tubes and were incubated at 5, 25 and 50˚C, for 410-days. Before the storage period, samples were analyzed regarding viscosity and density parameters. Over time, regularly, samples were withdrawn for mean droplet diameter, turbidity, and retention of encapsulated of αTOH determination.
(ii) *Simulation of cooking process:* 20 mL of the 9αTOH-NE freshly prepared one day before and stored overnight at 5 °C was poured in a 50 mL beaker (diameter 50 mm, height 60 mm). Then the sample was heated using a hot stirrer (As One, REXIM RSH—1DN, Osaka, JP) from 5 to 105 °C, with continuous stirring at 200 rpm up to the end of the cooking simulation. Once the cooking temperature reached 105 °C, the sample was boiled for 5 min. As a function of the time, the temperature was recorded using a thermometer. Along with the process, samples were withdrawn to analyze the amount of α-TOH retained into the nanoemulsion by gas-chromatography analysis. Considering the simulation of the cooking process, some portion of the water content was expected to evaporate.
(iii) *Effect of freeze-thawing:* one-cycle of freeze-thawing was evaluated by placing 8 mL of 9αTOH-NE (pH 7; 5 mM) in glass vials, which were incubated for 24 h at −18 °C, then followed by storage at 25˚C for another 24 h. After this test, the mean particle diameter was evaluated for stored 9αTOH-NE.
  After the solution condition’s treatments, the 9αTOH-NE were incubated at 25˚C for 24 h, and then analyzed for mean particle diameter and droplet charge.
(iv) *Solution condition*—*effect of pH:* 10 mL 9αTOH-NE was transferred to the beaker (20 mL), and the pH values (3–8) were adjusted using either 1 mol L^−1^ NaOH or 1 mol L^−1^ HCl.
(v) *Solution condition*—*effect of salt concentration:* 4 mL of 9αTOH-NE was mixed with 4 mL of ionic strength solution (NaCl) adjusted to different salt levels (e.g., 5, 25, 50, 75, 100, 125, 200, 300, 400, 500 mM).

### Physical characterization of the 9αTOH-NE

The dynamic viscosity and density were acquired using the Stabinger™Viscometer—SVM™3001 (Anton Paar, Austria). For the acquired values, the machine offers a reproducibility equal to 0.35%, falling in the range of 0.2 to 1000 mPa s.

(i) *Dynamic viscosity and density measurements*—*effect of α-terpineol fractions into the oil phase:* for the measurements, 2 mL of all α-TOH: SO fractions were injected, at 25˚C as reference temperature.
(ii) *Dynamic viscosity and density measurements*—*effect of storage temperature into the oil-in-water nanoemulsion:* for data acquisition, 2 mL of the 9αTOH-NE were analyzed under three different temperatures, fixed as 5, 25, 50˚C.
  The dynamic light scattering (DLS) technique using Zetasizer Nano ZS (Malvern Panalytical, Worcestershire, United Kingdom) was applied to determine the particle size and droplet charge, as a protocol adapted from Teo et al. (2016)^63^. To avoid multiple scattering effects, for both proceedings, the samples were diluted (1:100) before the analyses^66^. For pH and salt concentration effects, samples were diluted with the same level of pH or ionic strength solution^40^. For the freeze-thawing and thermal processing combined with the long-term storage test, the samples were diluted with buffer solution (pH 7, 5 mM).
(iii) *Particle size measurement:* for particle size determination, 2 mL of the diluted sample was added to a 12 mm square glass cuvette (PCS1115, 10 mm path length), and the measurements were done at 25˚C and 173° as backscattering angle. Particle size determination was expressed as the mean droplet diameter, polydispersity index, and particle size distribution. When it was convenient, one or more of these parameters were shown. A refractive index equal to 1.47 was adopted as a reference for the analysis.
(iv) *Droplet charge and conductivity measurements:* for electrophoretic mobility of oil droplets (reported as ζ-potential) and conductivity, the data were acquired using disposable folded capillary cells (DTS 1070), containing 1 mL of sample, and equilibrated at 25 ˚C for 60 s.
(v) *Turbidity measurements:* The turbidity measurements were taken using the Jasco V‐530 UV visible spectrophotometer (Tokyo, Japan). First, in a 50 mL-beaker 19.9 mL of water and 100 µL 9αTOH-NE were added (previously, the nanoemulsion was gently mixed for 10 s). Next, 2 mL of the diluted sample solution was placed in the glass cuvette (2 mm path length) and analyzed at 600 nm. Ultrapure water was used as a blank reference. The protocol here applied was an adaptation from Li & Lu (2016)^47^.

### Chemical degradation: retention of αTOH overtime into the 9αTOH-NE

(i) *Sample solution preparation:* first, the tube containing the 9αTOH-NE was gently shaken for 10 s for the sample’s homogenization. Then, in a 10 mL glass tube, containing 0.5 g of (NH4)_2_SO_4_ and 4 mL of methanol, 100 µL of the 9αTOH-NE was added. The glass tube containing the solution was vortexed for 1 min. Next, a clarification filtration step was conducted, where 2.0 mL of the solution was filtered through a 0.45 μm syringe filter. Finally, 1.5 mL of sample solutions were transferred to the injection vials for GC-analysis. The method here applied was a modification from Doost, Van Camp, Dewettinck, Van der Meeren (2019)^67^.
(ii) *Gas chromatography (GC) analysis: the* analysis was done using Shimadzu’s GC-2025 capillary gas chromatography (GC) system, equipped with a flame ionization detector (FID), AOC-20i as automatic liquid injector/sampler (10 µL syringe), and ZebronTMZB-WAXplusTM (7HG-G01311) as a polar column loaded with polyethylene glycol (PEG) as an active phase (30.0 m x 0.25 mm×0.25 µm). Helium was used as a makeup carrier gas with a fixed flow of 40 mL min^−1^. Temperature ramp from 80 °C (hold for 2.0 min) to 200 °C (hold for 5.0 min), with a heating rate as 20 °C min^−1^; then 200 °C to 80 °C (1.0 min), with cooling down rate of 40 °C min^−1^ was used a set-up condition of the column oven. 1 µL of the sample solution was injected (split 50:1; 3 mL min^−1^ purge flow) with the temperature fixed as 250 °C for both injector and detector. Chromatographic conditions herein used were followed Bicas, Fontanille, Pastore, Larroche (2010)^68^ as reference.
(iii) *Quantification of α-terpineol:* the quantification of α-TOH was done through a calibration curve (found in Supplementary Fig. 1(b)), where α-TOH ( ≥ 96% purity, Sigma-Aldrich) was used as standard. First, to confirm the identity of the α-TOH, the non-isothermal Kovats retention indices^69^ were determined for the PEG-column used in the whole GC-analysis. For this step, alkane standard-solution was used as a reference. Second, the calibration curve (n = 24) was built, varying the concentrations from 1.5 to 170 mg mL^−1^ as the inferior and superior limits, respectively. For the standard solution preparation, methanol was used as a solvent. For the curve structure, the peak ratio of the analyte *versus* the spiked concentration was used as a reference. Later, the limit of detection (LOD) and limit of quantification (LOQ) were determined as described by Shrivastava and Gupta (2011)^70^. To define the LOD and LDQ as well as to check the linearity of the analytical curve, a linear regression analysis was applied. Analysis of variance (ANOVA) at 95% of confidence-level was used. For both, α-TOH retention indices determination and calibration curve construction, the chromatographic conditions used were the same.

### Statical analysis

All the experiments were done as independent triplicate (*n* = 3), values are reported as mean ± standard deviation. When it was convenient and otherwise it was stated, one-way analysis of variance (ANOVA, *α* = 0.05) was applied, having Duncan Multiple Range Test (MRT) as *postdoc* test, using IBM^®^ SPSS^®^ as reference software.

### Reporting summary

Further information on research design is available in the Nature Research Reporting Summary linked to this article.

## Supplementary information

Supplementary Information

Reporting Summary

## Data Availability

Authors can confirm that all relevant data are included in the paper and/ or its supplementary information files.
