# Supplementary material for: Comprehensive study of α-terpineol-loaded oil-in-water (O/W) nanoemulsion: interfacial property, formulation, physical and chemical stability
Source: NPJ Sci Food. 2021 Nov 15;5:31. doi: 10.1038/s41538-021-00113-3 (PMC8593137; doi:10.1038/s41538-021-00113-3)
Supplement: Supplementary file 1 — Supplementary Information [file 41538_2021_113_MOESM1_ESM.pdf]

Supplementary information

(a)

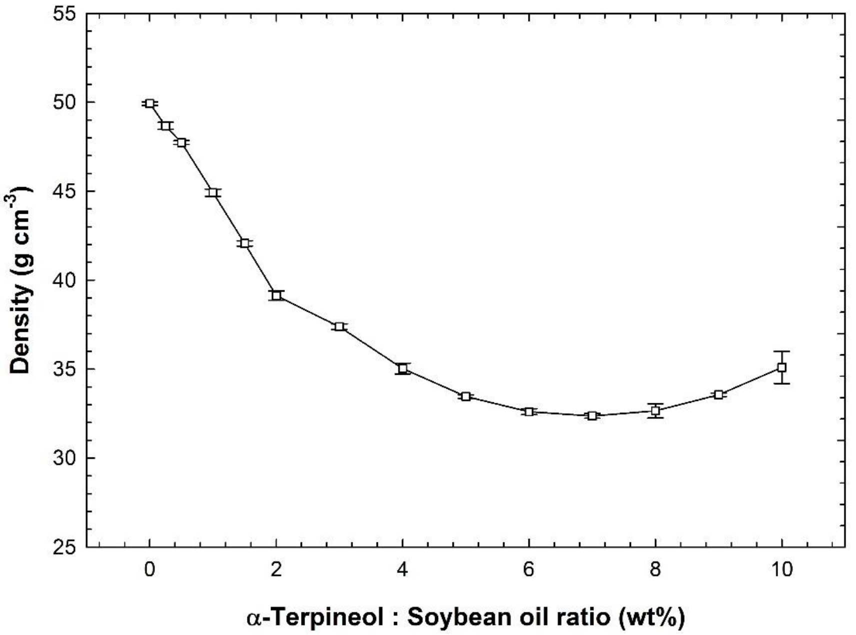

(b)

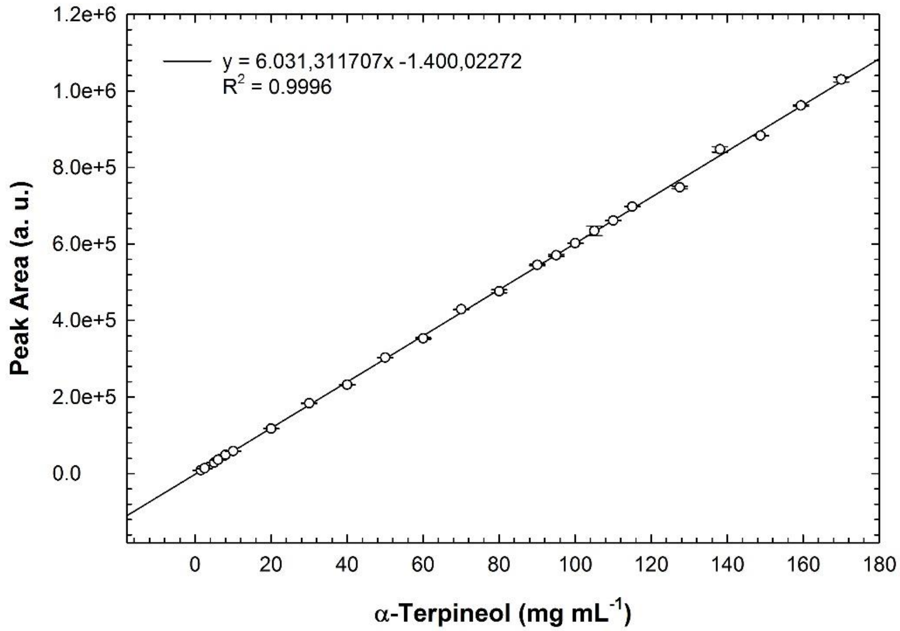

Supplementary Figure 1 – (a) Density values for the different ratios of α-terpineol: soybean oil used as reference for the measurements done for static interfacial tension (see Section 4.2.1). **(b)** Calibration curve obtained for the quantification of α-terpineol (see Section 4.9.3). Values are expressed here as n = 3; mean ± s.d.
